# Factors associated with glycemic control among adult patients with type 2 diabetes mellitus: a cross-sectional survey in Ethiopia

**DOI:** 10.1186/s13104-016-1896-7

**Published:** 2016-02-09

**Authors:** Tefera Kassahun, Tesfahun Eshetie, Hailay Gesesew

**Affiliations:** Dilchora Hospital, Dire Dawa, East Ethiopia; Department of Clinical Pharmacy, College of Health Sciences, Jimma University, Jimma, Ethiopia; Department of Epidemiology, College of Health Sciences, Jimma University, Jimma, Ethiopia; Discipline of Public Health, Faculty of Medicine, Nursing and Health Sciences, Flinders University, Adelaide, Australia

**Keywords:** Glycemic control, Fast blood glucose, Cross-sectional, Type 2 diabetic mellitus, Ethiopia

## Abstract

**Background:**

Even though the prevalence of type 2 diabetes mellitus is swelling rapidly in Ethiopia, data regarding glycemic control, a key strategy for marked reduction of diabetes mellitus complications, is scant. We have assessed the status of glycemic control and its contributing factors among adult patients with type 2 diabetes mellitus.

**Methods:**

This was a facility based cross-sectional survey of 325 adults with type 2 diabetes mellitus attending in Jimma University Teaching Hospital, South west Ethiopia. Data from all the patients were collected between February and April 2014. Glycemic level was assessed by using fasting blood glucose level, and ‘poor glycemic control’ was defined when fasting blood glucose level was above 130 mg/dL (7 mm/L). Analysis included both descriptive and inferential statistics, and SPSS version 20.0 was used for all analysis.

**Results:**

309 respondents were included in the survey. More than two-third (70.9 %) of the patients had poor blood glycemic control. Patients who were illiterate (AOR = 3.46, 95 % CI 1.01–11.91) and farmer (AOR = 2.47, 95 % CI 1.13–5.39) had high odds of poor glycemic control. In addition, taking combination of insulin and oral medication (AOR = 4.59, 95 % CI 1.05–20.14) and poor medication adherence (AOR = 5.08 95 % CI 2.02–12.79) associated statistically with poor glycemic control.

**Conclusion:**

Majority of patients had poor glycemic control. Patients with low level of education, being employed, on combinations of insulin and oral medication, and lower adherence to their medication were likely to have poor glycemic control. Education and awareness creation could be a cross cutting intervention for the significant factors.

## Background

Diabetes mellitus (DM) [1] has been ravaging millions of people from all over the world. Globally, diabetes has killed 4.6 million people in 2013 alone [2]. More than 77 % of morbidity [3] and 88 % of mortality [4] due to DM occur in low- and middle-income countries. In Ethiopia, the prevalence of diabetes was 3.5 % in 2011 [5]. Type 2 diabetes mellitus (T2DM) is the most common form of DM, accounting for more than 90 % of cases [2]. Control of diabetes is more than just taking medicine; other aspects of self-management such as self-monitoring of blood glucose, dietary restrictions, regular foot care and ophthalmic examination have all been shown to markedly reduce the incidence and progression of diabetes complications [2]. Previous studies have reported that suboptimal glycemic control could cost diabetes patients more care requirement, associated health-care costs, and loom the complications [6, 7].

The prevalence of poor glycemic control is paramount. A study done in Malaysia [8], 2015 showed that 72 % of patients had poor glycemic control, and two third of DM patients in Ethiopia [9] also had poor control. Previous studies assured that poor glycemic control correlated with enlarged risk of visual impairment [10], enlarged risk of kidney failure [11], and enlarged risk of cardiovascular disease [12]. In addition, the possible reasons included lack of awareness, time constraint, lack of adequate human power, poor adherence, and most importantly lack of appropriate guidelines and diabetes education for both care givers and patients [9, 13]. Therefore, it is easy to understand that adequate glycemic control among T2DM patients prevents short-term complications, decrease the risk of long-term complications, and decrease health care resource use and costs [14–16]. This figure indicted the need to give an attention for glycemic control. The *Healthy People 2020* aimed a 10 % reduction in the proportion of DM patients with poor glycemic control as a target [17].

But despite the swift growth of prevalence of T2DM in Ethiopia, data regarding glycemic control, the key strategy for marked reduction of acute and chronic complications of DM, is scant. Such data are noteworthy for the overall diabetic health care delivery services. We have assessed the status of glycemic control and its contributing factors among adult patients with T2DM.

## Methods

### Study design, settings and participants

**Fig. 1:**
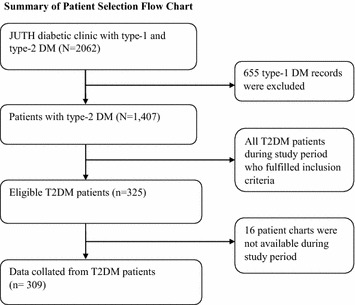
Summary of flowchart record selection, 2014

A facility based cross-sectional study was carried out in diabetic clinic at Jimma University Teaching Hospital (JUTH), Southwest Ethiopia from February 14 to April 9, 2014. The hospital serves the rural, urban and semi-urban areas. Drug Administration and Control Authority of Ethiopia Contents [18], a guideline similar with International Diabetes Federation clinical guideline [3] were followed for diagnosis and classification of DM. The study was conducted among T2DM adult patients (≥18 years) who were on active follow up for at least four visits. The required sample size was calculated via OpenEpi software using single population proportion calculation formula using the following assumptions: 58.2 % prevalence rate of poor glycemic control [19], 95 % confidence level, 5 % margin of error and 10 % non-response rate. Considering a correction formula, the total calculated sample yielded 325. Using sampling frame of DM records, simple random sampling technique was used to recruit the study participants (Fig. 1).

### Variables of the study and measurement

Glycemic level was the response variable that was coded as poor or good. Poor glycemic control was operationally defined if fasting blood glucose (FBG) level was above 130 mg/dl. Patients FBG reading for at least 4 months were recorded and computed the mean blood glucose level [20]. The explanatory variables included: socio-demographic and economic data (age, sex, level of education, marital status, occupation, income, ethnicity and religion), history of smoking, history of alcohol consumption, family history of DM, medication adherence, duration of therapy, body mass index (BMI) and number of diabetic medication.

Level of education was classified as illiterate (could not read and write their local language—‘Afan Oromo’ or ‘Amharic’), literate (could read and write but received no formal education), primary (received education up to class eight), secondary (received education class 9–12), and college/university (joined college or university). History of smoking and history of alcohol consumption has been assessed as during lifetime. Family history of DM was measured if any family member (mother or father) had DM. Morisky adherence score [21], eight-item yes/no questionnaire, was used to assess the self-reported measures of adherence to medications. For all questions, responses were coded 1 if patients responded ‘yes’ otherwise 0 if not, except one question that was coded reverse. The total score of adherence was classified into low adherence if the score was >2, medium adherence if between 1 and 2, and high adherence if 0.

Demographic and clinical characteristics were assessed via face-to-face interview and the average glycemic levels were reviewed from their chart. To ensure quality of data, the tool was developed in English, translated to local language (Amharic and Afan-Oromo) and back translated into English to check its consistency.

### Statistical analysis

Descriptive statistics included mean, median, standard deviations, and range values for continuous data; percentage and frequency tables for categorical data. Bivariate logistic regression analysis was conducted to see the existence of crude association and select candidate variables (with P value below 0.25 were considered) to multivariable logistic regression. We checked multi-collinearity among selected independent variables via variance inflation factor (VIF) and none was found. *P* value ≤0.05 was considered as a cut point for statistical significance in the final model. Fitness of goodness of the final model was checked by Hosmer and Lemeshow and was found fit. The Data was summarized using odds ratio (OR) and 95 % confidence interval. The analysis was done in Statistical Package for the Social Sciences (SPSS) 20 version software [22].

### Ethical considerations

Informed consent was obtained from study participants before the commencement of each interview, and no personal identification was registered. There was no any financial compensation or provision for the study participants. Permission was obtained from JUSH and the study was approved by institutional review board (IRB) of college of health sciences at Jimma University, Southwest Ethiopia.

## Results

### Socio-demographic and clinical characteristics of respondents

Three hundred and twenty-five DM patients were considered eligible, whereof 16 were excluded since their chart were not available (Fig. 1). In total, 309 patients included in the analysis making 95 % response rate. Table 1 shows demographic and economic characteristics of the respondents. Males were over-represented (61.8 %) and almost two-fifth (36.9 %) of the respondents represented the age group 51–60 years. Nearly half (46.6 %) of the respondents followed Muslim religion and four out of five (81.2 %) respondents were married. Two fifth (36.2 %) respondents attained grades (1–8), and 30.4 % of respondents were farmers.

**Table 1:** Socio-demographic characteristics of T2DM patients on follow up at JUTH, 2014

Socio-demographic characteristics (n = 309)	Categories	n (%)
Sex	Male	189 (61.8)
	Female	120 (38.2)
Age	18–32	5 (1.6)
	33–41	30 (9.7)
	42–50	72 (23.3)
	51–60	114 (36.9)
	≥61	88 (28.5)
Marital status	Married	251 (81.2)
	Single	9 (2.9)
	Divorce	12 (3.9)
	Widow/er	37 (12.0)
Ethnicity	Oromo	170 (55.1)
	Amhara	78 (25.2)
	Keficho	21 (6.8)
	Gurage	10 (3.2)
	Dawero	8 (2.6)
	Yem	8 (2.6)
	Other^a^	14 (4.5)
Educational level	Illiterate	87 (28.2)
	Read & write	22 (7.1)
	1–8	112 (36.2)
	9–12	44 (14.3)
	College/University	44 (14.2)
Religion of respondents	Muslim	144 (46.6)
	Orthodox	138 (44.7)
	Protestant	23 (7.4)
	Others^b^	4 (1.3)
Occupation	Farmer	94 (30.4)
	No job	78 (25.2)
	Employed	72 (23.3)
	Merchant	29 (9.4)
	Daily labor	36 (11.7)

^a^Tigre, Wolayita

^b^Catholic, Jehovah witness

Table 2 shows clinical characteristics of the respondents. Mean BMI of the respondents was 24.4(±4.39) kg/m^2^, and 33 % of the respondents had overweight. Great majority of the respondents had not access for self-monitoring blood glucose (SMBG). About half of the respondents had not get social support, and one fourth (24.6 %) of the respondents had family history of DM. The prevalence of smoking and alcohol, respectively, was 3 and 5 %. DM patients were followed for an average of 7.2 (±5.8) years with a minimum of 4 months and a maximum of 40 years. Diabetic neuropathy was the most common DM complication accounting for 41.5 %. Regarding diabetic medications, 63.1 % of respondents were taking oral medication only followed by insulin (25.6 %). The respondents took a mean of 3.66 (±1.60) medications ranged between 1 and 7 medications.

**Table 2:** Clinical characteristics of T2DM patients on follow up at JUTH, 2014

Clinical characteristics (n = 309)	Category	n (%)
Body mass index	Under weight	17 (5.5)
	Normal weight	161 (52.1)
	Over weight	102 (33.0)
	Obese	29 (9.4)
Family/social support	Yes	175 (56.6)
	No	134 (43.4)
Family history	Yes	76 (24.6)
	No	233 (75.4)
Habit of smoking	Smoker	9 (2.9)
	Non-smoker	287 (92.9)
	Ex-smoker	13 (4.2)
Alcohol drinking	Non-drinker	293 (94.8)
	Drinker	16 (5.2)
Access for self-monitoring blood glucose	Yes	30 (9.7)
	No	279 (90.3)
Duration of treatment	<5	145 (46.9)
	5–10	105 (33.9)
	>10	59 (19.2)
Anti-diabetics	Insulin	79 (25.6)
	Oral medication	195 (63.1)
	Insulin & oral medication	35 (11.3)
Diabetic complication (n = 352)	Neuropathy	146 (41.5)
	Nephropathy	56 (15.9)
	Retinopathy	94 (26.7)
	Cardiac complications	56 (15.9)
No. of medications	<2 drugs	115 (37.2)
	2–4 drugs	135 (43.7)
	>4 drugs	59 (19.1)

### Glycemic control level and its contributing factors among T2DM patients

Poor glycemic control was seen in 219 (70.9 %) respondents. Age, sex, education, occupation, BMI, anti-diabetics, number of medications and adherence to medication were the candidate variables for multiple logistic regression. The following factors were statistically significant in bivariate logistic analysis: being illiterate, being achieved grades 1–8, being employed, being farmer, being under weight, taking combinations of insulin and oral medication, taking 2–4 drugs and >4 drugs, medium medication adherence and low medication adherence. In multiple logistic regression, statistically significant difference was found in poor glycemic control to education, occupation, anti-diabetics and level of medication adherence.

Table 3 presents the multiple logistic regression analysis with demographic and clinical characteristics, and poor glycemic control. The association of poor glycemic control among illiterate respondents was three times (AOR = 3.46, 95 % CI 1.01–11.92) greater than among those who were in college/university. The relative probability of poor glycemic control among farmers was higher than (AOR = 2.47, 95 % CI 1.14–5.39) unemployed ones. Compared to respondents who took oral medication, respondents who took combinations of insulin and oral medication were five times (AOR = 4.59, 95 % CI 1.05–20.14) more likely to have poor glycemic control. On the other hand, the odds of poor glycemic control among patients who had medium and low adherence to their medication were three (AOR = 3.49, 95 % CI 1.72–7.09) and five times (AOR = 5.08, 95 % CI 2.02–12.79) more than patients who had high adherence to their medication respectively.

**Table 3:** Factors independently associated with poor glycemic level among T2DM patients JUTH, 2014

Variables (n = 309)	Glycemic level		Crude OR (95 % CI)	Adjusted OR (95 % CI)	P value
	Good, n (%)	Poor, n (%)			
Education					
Illiterate	18 (20.7 %)	69 (79.3 %)	2.65 (1.20–5.87)^a^	3.46 (1.01–11.91)	0.049^a^
Read & write	10 (45.5 %)	12 (54.5 %)	0.83 (0.29–2.33)	0.81 (0.20–3.26)	0.766
1–8	31 (27.7 %)	81 (72.3 %)	1.81 (0.87–3.75)	2.45 (0.85–7.03)	0.095
9–12	13 (29.5 %)	31 (70.5 %)	1.65 (0.68–3.99)	1.97 (0.69–5.55)	0.202
College/University	18 (40.9 %)	26 (59.1 %)	1	1	
Occupation					
No job	31 (39.7 %)	47 (60.3 %)	1	1	
Employed	21 (29.2 %)	51 (70.8 %)	1.60 (0.81–3.16)	2.65 (0.96–7.24)	0.048^a^
Merchant	6 (20.7 %)	23 (79.3 %)	2.53 (0.92–6.91)	2.69 (0.86–8.37)	0.086
Farmer	19 (20.2 %)	75 (79.8 %)	2.60 (1.32–5.12)^a^	2.47 (1.13–5.39)	0.023^a^
Daily labor	13 (36.1 %)	23 (63.9 %)	1.17 (0.51–2.64)	2.22 (0.80–6.11)	0.124
Anti-diabetics					
Insulin	21 (26.6 %)	58 (73.4 %)	1.38 (0.77–2.47)	1.77 (0.60–5.19)	0.298
Oral medication	65 (33.3 %)	130 (66.7 %)	1	1	
Insulin and oral medication	4 (11.4 %)	31 (88.6 %)	3.88 (1.31–11.45)^a^	4.59 (1.05–20.14)	0.043^a^
Medication adherence					
High	52 (45.2 %)	63 (54.8 %)	1	1	
Medium	25 (21.4 %)	92 (78.6 %)	3.04 (1.71–5.43)^a^	3.49 (1.72–7.09)	0.001^a^
Low	13 (16.9 %)	64 (83.1 %)	4.06 (2.02–8.18)^a^	5.08 (2.02–12.79)	0.001^a^

^a^Statistically significant at P value <0.05

## Discussion

The main goal of diabetes management is to ensure optimal glycemic control. We have assessed the magnitude of poor glycemic control and associated factors among T2DM patients. Results of this study showed that nearly three fourth of patients with T2DM had poor glycemic control. This result was comparable to those obtained in an earlier study that reported 65 % [23] and 81.9 % [24] of respondents had poor glycemic control. This significant proportion of poor glycemic control in the country underpins the need to work more on self-management strategies of DM patients. The current finding is far higher than from developed countries such as 12.9 % in United States [7]. This variation could be due to knowledge difference of respondents between developing and developed countries, absence of uniform guidelines in assessing glycemic control for physicians to set the score cutoff, and the presence health insurance and the difference in health insurance coverage and access to primary care [7, 25, 26].

Significant difference of poor glycemic control was observed among illiterates than college/university graduates. Consistent with this, studies from Jordan [27] and China [28] reported the correlation of lower level of education and poor glycemic control. This could indicate illiterate patients had low diabetes knowledge, low self-management behaviors, lower self-efficacy and lower continuity of care. Thus, we are recommending investment on getting rid of illiteracy as it has a significant impact on the reduction of diabetic morbidity and mortality [29, 30]. Poor glycemic control appeared to be greater among farmers compared to unemployed respondents. This, however, might be due to allocating less time for self-management. Majority of the farmers in this study were also illiterate compared to unemployed respondents implying that farmers might have poor awareness of self-care of DM.

It was also likely that the difference in glycemic control might result from differences in anti-diabetic treatment. Compared to respondents on oral medication, respondents who were on combinations of insulin and oral medication were five times more likely to have poor glycemic control. This association was corroborated by study done in Malaysia [31]. The poor control among patients receiving a combination of insulin and oral anti-diabetic drugs shows that multi therapy might challenge satisfactory glycemic control. There was a strong inverse association between adherence level and poor glycemic control as supported by the previous studies [32]. Showing very poor outcomes among respondents who fail to comply with the prescribed clinical regimen is not surprising [33]. Accordingly, counseling and improving adherence rather than changing medication or altering the dose has been suggested [33]. It is plausible to have an endeavor to tackle non-adherence by designing strategies encompassing cost, health belief, dosing frequency, personality disorders and patient provider relationship.

Worth noting limitations should have noted in this study. The use of FBS over HbA1c my under estimate the prevalence of poor glycemic control even though FBS was found more reliable than HbA1c [34]. The institutional based nature of the study might not infer for other diabetic patients. The nature of cross-sectional study design does not show temporal relationship or causality. Recall bias might also be there during measuring self-report of medication to adherence.

## Conclusion

In summary, the findings from the current study agree in many points with the findings of previous publications. Significant number of DM patients did not achieve the recommended glucose level. This was affected by education, occupation, anti-diabetic treatment and adherence status of the patients. Education and awareness creation could be a cross cutting intervention for the significant factors. We recommend further population based research.

## Abbreviations

BMI: body mass index
DM: diabetes mellitus
FBG: fasting blood glucose
HbA1c: glycosylated hemoglobin
IDFA: International Diabetes Federation Atlas
JUSH: Jimma University Specialized Hospital
OHA: oral hypoglycemic agent
SMBG: self-monitoring blood glucose
SDSCA: summary of diabetes self-care activities
T2DM: type-2 diabetes mellitus
